# Do Smokers’ Perceptions of the Harmfulness of Nicotine Replacement Therapy and Nicotine Vaping Products as Compared to Cigarettes Influence Their Use as an Aid for Smoking Cessation? Findings from the ITC Four Country Smoking and Vaping Surveys

**DOI:** 10.1093/ntr/ntac087

**Published:** 2022-04-03

**Authors:** Hua-Hie Yong, Shannon Gravely, Ron Borland, Coral Gartner, K Michael Cummings, Katherine East, Scott Tagliaferri, Tara Elton-Marshall, Andrew Hyland, Maansi Bansal-Travers, Geoffrey T Fong

**Affiliations:** Department of Psychology, Deakin University, Geelong, VIC, Australia; Department of Psychology, University of Waterloo, Waterloo, ON, Canada; Melbourne Centre for Behaviour Change, School of Psychological Sciences, The University of Melbourne, Melbourne, VIC, Australia; School of Public Health, The University of Queensland, Brisbane, QLD, Australia; Department of Psychiatry & Behavioral Sciences, Medical University of South Carolina, Charleston, SC, USA; Department of Psychology, University of Waterloo, Waterloo, ON, Canada; National Addiction Centre, Institute of Psychiatry, Psychology and Neuroscience, King’s College London, London, UK; Department of Psychology, Deakin University, Geelong, VIC, Australia; School of Epidemiology and Public Health, University of Ottawa, Ottawa, ON, Canada; Department of Health Behavior, Roswell Park Comprehensive Cancer Center, Buffalo, NY, USA; Department of Health Behavior, Roswell Park Comprehensive Cancer Center, Buffalo, NY, USA; Department of Psychology, University of Waterloo, Waterloo, ON, Canada; Ontario Institute for Cancer Research, MaRS Centre, Toronto, ON, Canada

## Abstract

**Introduction:**

This study examined whether smokers’ harm perceptions of nicotine replacement therapy (NRT) and nicotine vaping products (NVPs) relative to cigarettes predicted their subsequent use as smoking cessation aids during their last quit attempt (LQA).

**Aims and Methods:**

We analyzed data from 1,315 current daily smokers (10+ cigarettes per day) who were recruited at Wave 1 (2016), and who reported making a quit attempt by Wave 2 (2018) of the International Tobacco Control Four Country Smoking and Vaping Surveys in Australia, Canada, England, and the United States. We used multinomial logistic regression models to examine prospective associations between harm perceptions of (a) NRT and (b) NVPs and their use at LQA, controlling for socio-demographic and other potential confounders.

**Results:**

Smokers who perceive that (a) NRT and (b) NVPs are much less harmful than cigarettes were more likely to subsequently use the respective product as an aid than using no aid or other aids during LQA (adjusted relative risk ratio [aRRR] = 3.79, 95%CI = 2.16–6.66; and aRRR = 2.11, 95%CI = 1.29–3.45, respectively) compared to smokers who perceive these products as equally or more harmful. Additionally, those who perceive NVPs as much less harmful than cigarettes were less likely to use NRT as a quit aid (aRRR = 0.34, 95%CI = 0.20–0.60). No country variations for these associations were found.

**Conclusions:**

This study found that smokers’ perceptions of the harmfulness of (a) NRT and (b) NVPs relative to cigarettes predicted the respective product use when trying to quit smoking. Corrective education targeting misperceptions of nicotine products’ relative harmfulness may facilitate their use for smoking cessation.

**Implications:**

Nicotine replacement therapy and nicotine vaping products are two commonly used smoking cessation aids. This study demonstrates that misperceptions of the harms of nicotine products relative to cigarettes influence their use for smoking cessation. Believing that nicotine vaping products are much less harmful than cigarette smoking may lead some smokers to prefer these products over nicotine replacement therapy to aid smoking cessation. Education targeting misperceptions of nicotine products’ harmfulness relative to cigarettes may enable smokers to make informed choices about which are appropriate to aid smoking cessation.

## Introduction

There is a continuum of risk across different nicotine products.^1^ Combustible tobacco products (eg, cigarettes) are the most harmful to health, while nicotine replacement therapy (NRT) products are the least harmful. While long-term epidemiological evidence is not available for nicotine vaping products (NVPs, known as e-cigarettes), toxicological evidence suggests their risk profile is likely to be somewhere in between cigarettes and NRT, but closer to the NRT level.^1–4^

Both NRT and NVPs are often used as smoking cessation aids.^5^ In many countries, NRT is a government-approved medical therapy for smoking cessation and is recommended in clinical practice guidelines as an effective cessation aid.^6,7^ Clinical trials have consistently demonstrated the safety and efficacy of NRT for smoking cessation.^8^ Smokers who quit with the help of NRT have 50%–60% greater likelihood of succeeding than those who try to quit without using an aid.^8^ However, the real-world effectiveness of NRT for smoking cessation appears lower than that found in clinical trials,^9^ which has been attributed to the way it is used in real life (eg, under-dosing and premature discontinuation of treatment).^10^ Past research has found widespread misperception of NRT harmfulness.^11–13^ Research studies have also found misperception of NRT harmfulness was associated with lower likelihood of using NRT in past quit attempts, lower consideration of using NRT for future quit attempts and lower compliance among those who used NRT during quit attempts.^14,15^ The findings suggest that many smokers are misinformed about the health harms of NRT and these misperceptions not only undermine NRT uptake but also its proper utilization for smoking cessation. Other research suggests that uncontrolled recall bias may also account for the lower effectiveness of NRT for smoking cessation often observed in real-world studies.^16^ Nevertheless, these past findings were based mainly on cross-sectional studies precluding the ability to determine the directionality of effect as use could also affect perception. Prospective cohort studies are needed to better understand how perceptions of NRT harmfulness would influence its use for smoking cessation in the real-world setting.

NVP use has increased rapidly in the last decade.^3^ Smoking cessation is one of the reasons smokers use NVPs.^17^ However, their effectiveness for smoking cessation is debated. The latest Cochrane systematic review provided moderate certainty evidence to indicate that NVPs are more effective than NRT for smoking cessation.^18^ Like NRT, harm perceptions of NVPs may impact the extent of NVP use. A growing body of literature suggests that misperceptions of the harmfulness of NVPs are substantial and increasing over time.^19,20^ Studies have also found that smokers who believe that NVPs are less harmful than smoking are more likely to use them than their counterparts who believe otherwise.^20–22^ However, it is unclear as to what extent the association between perceptions and use behavior applies to smoking cessation specifically. More studies are needed to understand how harm perceptions of NVPs influence its use for smoking cessation.

Recent research indicates that misperceptions of the harmfulness of nicotine are widespread^23,24^ and they contribute to misperceptions of NRT and NVPs.^13^ This may account for the high correlation found between harm perceptions of NRT and NVPs.^25^ Regardless of the mechanisms, harm perceptions are likely to generalize across all nicotine products, rather than be product specific. This cross-product relationship between harm perceptions of one nicotine product (eg, NRT) and use of other nicotine products (eg, NVPs), particularly for the purpose of smoking cessation, has not been studied before.

The regulatory context of a country has been shown to influence the extent of use of nicotine products.^21^ Differences in regulatory environments across countries also appear to affect harm perceptions of nicotine products.^26,27^ Access, availability and policy for NRT and NVPs vary across the countries studied here.^28^ For example, NRT is rarely accessed via prescription in Australia, Canada, and the United States, but is common in England.^29,30^ The regulatory environment for NVPs is more restrictive in Australia, where retail sales and marketing are banned and legal use requires a doctor’s prescription.^31^ In contrast, in England, the sale of NVPs is legal and their use for smoking cessation is supported, both by Public Health England and the National Institute for Health and Care Excellence (NICE) clinical practice guidelines.^32^ During data collection for our study (2016 and 2018), NVPs were not legal for sale in Canada, but were widely accessible due to poor enforcement of sales bans in retail stores.^33^ In the United States, NVPs were legal, but were not promoted for cessation by public health entities. While there is some evidence to suggest that regulatory environments influence harm perceptions of nicotine-containing products,^26,27^ further research is warranted to understand how regulations around NRT and/or NVPs would influence harm perceptions of these nicotine products and in turn, the extent of their use for smoking cessation.

**Figure 1.**
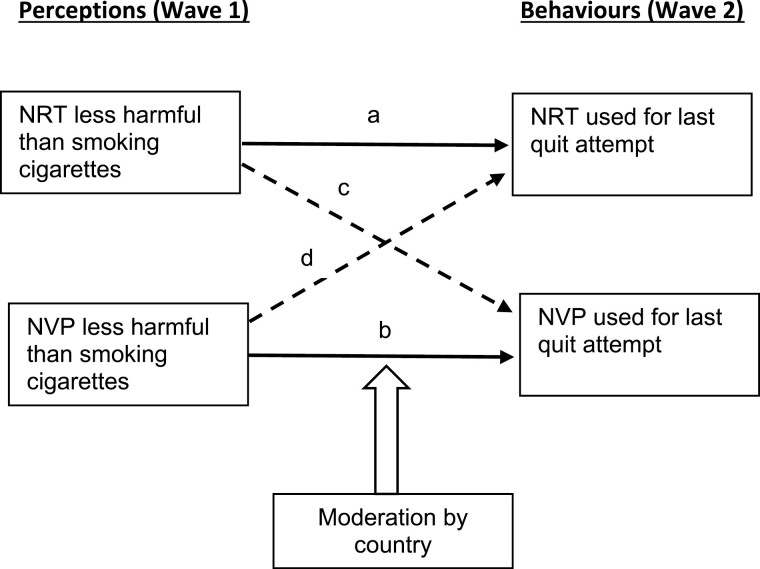
Hypothesized relationships of baseline harm perceptions with subsequent use behavior within and between products.

This prospective cohort study aimed to investigate: (1) how harm perceptions of NRT and NVPs among heavy daily smokers predicted their subsequent use during their last quit attempt (LQA); (2) whether harm perceptions of one nicotine product would predict use of the alternative product during LQA; and (3) whether country would moderate the above perception-behavior associations. As depicted in Figure 1, we hypothesized that accurate (ie, less harmful) perceptions of NRT or NVPs would predict a higher rate of use of the respective product as well as the alternate product during LQA; and that country would moderate the predictive associations with no clear expectation of how they might differ between countries.

## Methods

### Sample and Design

We analyzed data from Waves 1 (2016) and 2 (2018) of the ITC Four Country and Vaping (ITC 4CV) Surveys, a cohort study consisting of four parallel online surveys conducted in Canada, the United States, England, and Australia. In addition to respondents retained from the ITC Four Country Survey (the predecessor of ITC 4CV), adults (≥ 18 years) were recruited by commercial panel firms in each country at Wave 1 (July–November 2016) as cigarette smokers (smoked at least 100 cigarettes in their lifetime), recent ex-smokers (quit within ≤2 years), or at-least-weekly NVP users (vapers). All Wave 1 respondents were invited to complete the Wave 2 survey (February–July 2018). The sample in each country was designed to be as representative as possible of smokers and vapers and used either probability-based sampling frames or nonprobability quota samples. Details of the conceptual framework and the survey methodology can be found in Fong et al.^34^ and Thompson et al.,^35^ and in the technical reports.^36,37^

We limited the analytical sample for this analysis to current cigarette smokers (regardless of whether they used any NVPs or not), who reported smoking at least 10 cigarettes per day in 2016, made a quit attempt by the 2018 follow-up, and completed both the 2016 and 2018 surveys (see Supplementary Figure S1). Overall, 1,315 individuals met these criteria across the four countries in Canada (*n* = 461), United States (*n* = 219), England (*n* = 353), and Australia (*n* = 282).

### Measures

The survey questionnaires, with original response options, can be found at the ITC Project website: https://itcproject.org/surveys/. The following variables were used in the current study:

#### Outcomes:

Quit aids used during last quit attempt (LQA) were assessed by asking participants who had made a quit attempt at the follow-up survey (Wave 2) with the following questions: (1) if they had used any type of nicotine replacement product, such as patches, gum, or mouth spray, at the time of their last (smokers) or current (ex-smokers) quit attempt (referred to hereafter as last quit attempt [LQA]) (Yes/No); (2) if they had used a vaping product on their last/current quit attempt (Yes/No); and (3) if they had used any other aids/assistance which included stop-smoking medications (e.g., varenicline, bupropion), smokeless tobacco products (heated tobacco product, snus), stop-smoking service (eg, quitline, behavior therapy), online cessation help (eg, apps, website, support groups), self-help materials (pamphlets, brochures). Based on their responses to these survey questions, an outcome variable with four mutually exclusive categories was derived: (i) “*No/other quit aids”;* (ii) “*Any use of NRT alone or with other aids excluding NVP”*; (iii) “*Any use of NVP alone or with other aids excluding NRT”*; and (iv) “*Any use of both NRT and NVP excluding prior categories”*.

### Baseline Measures

#### Predictor variables:

Perceptions of harm of NRT and NVP relative to cigarettes were assessed using the following respective questions: *“Compared to smoking cigarettes, how harmful do you think nicotine replacement products are? Nicotine replacement products include patches, gum, inhalers, mouth spray, and various other nicotine products that have been approved as medicines”* and “*Compared to smoking cigarettes, how harmful do you think vaping (using e-cigarettes) is?”* The original response options for both questions were: “much less harmful”, “somewhat less harmful”, “equally harmful”, “somewhat more harmful”, “much more harmful” or “don’t know”. For analysis purposes, responses to the first two response options were treated as accurate perceptions and the rest were treated as inaccurate perceptions. The “equally harmful”, “somewhat more harmful” and “much more harmful” categories were combined to ensure sufficient cases for analysis as the reference category. Both measures had been shown to work well in cognitive testing^38^ and past research.^25^

#### Covariates:

Age in years (18–25, 25–39, 40–54, or 55+), gender (male or female), education (low, moderate or high), income (low, moderate, high or not reported), ethnicity (ethnic majority or minority), country (Canada, United States, England, or Australia), beliefs about nicotine harmfulness (not at all through to extremely harmful), and knowledge of smoking health effects (derived using four items assessing whether respondents believed smoking causes strokes, blindness, breast cancer, or mouth cancer, and the affirmative responses were summed to give a total score ranging from 0 to 4).

### Data Analysis

All statistical analyses were performed in Stata version 16 (StataCorp, TX, USA). Sample characteristics were examined using frequencies and (unweighted) percentages for categorical variables, and mean and standard deviations for continuous variables, separately by country. Multinomial logistic regression was employed to examine the association of daily smokers’ harm perceptions of nicotine products (ie, NRT and NVP) relative to cigarettes (much less harmful, somewhat less harmful or do not know vs. equally/more harmful) at Wave 1 (baseline) with choice of products used as a quit aid at their LQA initiated by Wave 2 (follow-up; ie, no/other aids as a reference compared with use of NRT, NVP or both products, while adjusting for the covariates listed above). Predictor by country interaction terms were added into the model to test for whether effects varied across countries. To explore the specificity of the predictive effect of these two product harm perceptions on choice of quit aid used for LQA, additional analyses were conducted using the outcome variable: no quit aids at LQA as the reference to compare with use of any nicotine products (which included a small number of heated tobacco products and smokeless tobacco products reported, *n* = 22) and use of only non-nicotine products as quit aids for LQA. Relative risk ratios (RRR) and 95% confidence intervals (CI) were estimated. Statistical significance was set to an alpha of 0.05.

## Results

### Sample Characteristics and Harm Perceptions

The unweighted baseline sample characteristics of smokers and their perceptions of the harmfulness of nicotine, NRT and NVPs are presented in Table 1. Overall, only 13% perceive that nicotine is either not at all or slightly harmful for health, 61% perceive NVPs as less harmful than cigarettes, and 76% perceive NRT as less harmful than cigarettes. Notably, the percentage who reported that NRT is less harmful than cigarettes was highest in England and lowest in the United States (79% and 71%, respectively), with similar country differences for NVPs being less harmful (67% and 54%, respectively). As seen in Table 1, one in five smokers in our sample (20%) reported using NRT (either exclusively or in combination with other aids) during their LQA, around one in four (26%) reported using NVP (either exclusively or in combination with other aids), and around 12% reported using both products (exclusively or in combination) during LQA, with the majority reporting using no aid (29%) and the rest using some other aids during their LQA (13%).

**Table 1.** Sample Characteristics of Baseline (Wave 1) Current Daily (10+ cigs per day) Smokers* and Quit Aids used at Last Quit Attempts (Wave 2) by Country

Variables	Canada *N* = 461	United States n=219	England n=353	Australia n=282	Total n=1,315
W1 Age Group, *n* (%)					
** **18–24	37 (8)	13 (6)	31 (9)	5 (2)	86 (7)
** **25–39	109 (24)	31 (14)	57 (16)	44 (16)	241 (18)
** **40–54	150 (33)	63 (29)	119 (34)	106 (38)	438 (33)
** **55+	165 (36)	112 (51)	146 (41)	127 (45)	550 (42)
W1 Gender, *n* (%)					
** **Male	219 (48)	100 (46)	163 (46)	132 (47)	614 (47)
** **Female	242 (52)	119 (54)	190 (54)	150 (53)	701 (53)
W1 Education, *n* (%)					
** **Low	160 (35)	76 (35)	131 (38)	110 (39)	477 (37)
** **Moderate	213 (47)	96 (44)	123 (35)	110 (39)	542 (42)
** **High	84 (18)	47 (21)	95 (27)	80 (22)	286 (22)
** **No answer	4 (0)	0 (0)	4 (0)	2 (0)	10 (0)
W1 Income, n (%)					
** **Low	173 (38)	77 (35)	81 (23)	96 (34)	427 (32)
** **Moderate	130 (28)	67 (31)	162 (46)	71 (25)	430 (33)
** **High	123 (27)	73 (33)	86 (24)	101 (36)	383 (29)
** **No information	35 (8)	2 (1)	24 (7)	14 (5)	75 (6)
W1 Ethnicity, *n* (%)					
** **Ethnic majority	410 (90)	185 (84)	332 (95)	267 (95)	1194 (92)
** **Ethnic minority	41 (9)	34 (16)	14 (3)	15 (5)	104 (7)
** **Do not know	10 (1)	0 (0)	7 (1)	0 (0)	17 (1)
W1 Knowledge of smoking harms (0–4) Mean (SD)	2.8 (1.1)	2.3 (1.2)	2.5 (1.1)	2.7 (1.2)	2.6 (1.2)
W1 Belief of nicotine being harmful to health, *n* (%)					
** **Not at all	9 (2)	3 (1)	21 (6)	8 (3)	41 (3)
** **Slightly	35 (8)	25 (11)	51 (14)	19 (7)	130 (10)
** **Moderately	119 (26)	59 (27)	79 (22)	83 (29)	340 (26)
** **Very	185 (40)	70 (32)	100 (28)	92 (33)	447 (34)
** **Extremely	105 (23)	58 (26)	92 (26)	73 (26)	328 (25)
** **Do not know	8 (2)	4 (2)	10 (3)	7 (2)	29 (2)
W1 Belief of NRT harmfulness relative to cigarettes, *n* (%)					
** **Much Less	163 (35)	71 (32)	137 (39)	120 (43)	491 (37)
** **Somewhat Less	183 (40)	86 (39)	142 (40)	99 (35)	510 (39)
** **Equally	57 (12)	29 (13)	25 (7)	28 (10)	139 (11)
** **More	13 (3)	6 (3)	10 (3)	7 (2)	36 (3)
** **Do not know	45 (10)	27 (12)	39 (11)	28 (10)	139 (11)
W1 Belief of NVP harmfulness relative to cigarettes, *n* (%)					
** **Much Less	91 (20)	38 (17)	91 (26)	52 (18)	272 (21)
** **Somewhat Less	188 (41)	81 (37)	145 (41)	114 (40)	528 (40)
** **Equally	91 (20)	50 (23)	59 (17)	45 (16)	245 (19)
** **More	20 (4)	10 (5)	18 (5)	7 (2)	55 (4)
** **Do not know	71 (15)	40 (18)	40 (11)	64 (23)	215 (16)
W2 Nicotine product used as quit aids at LQA, n (%)					
** **No aid or other aids	177 (38)	115 (53)	121 (34)	140 (50)	553 (42)
** **NRT only ^a^	103 (22)	36 (16)	46 (13)	74 (26)	259 (20)
** **NVP only ^b^	102 (22)	45 (21)	147 (41)	42 (15)	336 (26)
** **Both NRT and NVP^c^	79 (17)	22 (10)	40 (11)	26 (9)	167 (12)
W2 Quit aids used at LQA, n (%)					
** **No aids	118 (26)	82 (37)	91 (26)	86 (31)	377 (29)
** **Any nicotine aids^^^	286 (62)	105 (48)	233 (66)	42 (50)	766 (58)
** **Only non-nicotine aids	57 (12)	32 (15)	29 (8)	54 (19)	172 (13)

NB. Percentages and means are unweighted;

Among those who have made a quit attempt by Wave 2 (2018);

NRT = nicotine replacement therapy; NVP = nicotine vaping product; W1 = Wave 1; W2 = Wave 2; LQA = last quit attempt;

NRT only defined as any use of NRT either exclusively or in combination with other aids but exclude NVP for LQA.

NVP only defined as any use of NVP either exclusively or in combination with other aids but exclude NRT for LQA.

Both NRT and NVP defined as use of both products for LQA either exclusively or in combination with other aids.

Include any use of heated tobacco products [HTP] and smokeless tobacco (asked only in Canada and the US).

### Association of NRT and NVP Harm Perceptions Relative to Cigarettes with Own-product Use as a Quit Aid during LQA

Results from the multinomial logistic regression are presented in Table 2 (see Supplementary Table S1 for bivariate associations and Supplementary Table S2 for full details). After adjusting for covariates, smokers were more likely to use NRT as a quit aid for their LQA relative to use of other aids or no aids if they perceive NRT is much less harmful (adjusted relative risk ratio [aRRR] = 3.79, 95% confidence interval [CI] = 2.16–6.66), or somewhat less harmful (aRRR = 1.98, 95% CI = 1.15–3.42) compared to equally/more harmful than cigarettes (ie, approximately four times and two times as likely to do so, respectively).

**Table 2.** Prospective Association Between Wave 1 Nicotine Product Harm Perceptions and Wave 2 Choice of Nicotine Product used as an Aid for Last Quit Attempts Among Baseline Daily Smokers Smoking 10+ Cigarettes per day who had made a Quit Attempt by Wave 2 (*N* = 1289^^^).

Wave 1 Predictors	Wave 2 NRT use vs other/no aids ^a^ aRRR (95% CI)	Wave 2 NVP use vs other/no aids ^b^ aRRR (95% CI)	Wave 2 Both NRT & NVP use vs other/no aids ^c^ aRRR (95% CI)
NRT Relative Harm Perception			
Much Less harmful	**3.79 (2.16, 6.66)** ^***^	1.51 (0.88, 2.61)	**1.96 (1.03, 3.73)** ^*^
Somewhat Less harmful	**1.98 (1.15, 3.42)** ^*^	1.47 (0.89, 2.05)	1.16 (0.62, 2.15)
Equal/More harmful	*Reference*	*Reference*	*Reference*
Do not know	0.98 (0.45, 2.11)	1.69 (0.85, 3.36)	0.82 (0.29, 2.25)
NVP Relative Harm Perception			
Much Less harmful	**0.34 (0.20, 0.60)** ^***^	**2.11 (1.29, 3.45)** ^**^	1.29 (0.71, 2.35)
Somewhat Less harmful	0.69 (0.46, 1.05)	1.34 (0.88, 2.05)	1.20 (0.72, 2.01)
Equal/More harmful	*Reference*	*Reference*	Reference
Do not know	0.64 (0.38, 1.07)	**0.53 (0.29, 0.96)** ^*^	**0.32 (0.14, 0.75)** ^**^

Note: NRT = Nicotine Replacement Therapy, NVP = Nicotine Vaping Products;

aRRR = adjusted Relative Risk Ratio which estimates the likelihood of the outcome (e.g., NRT use vs other/no aids) for a variable, holding all other variables in the model constant; CI = Confidence Intervals;

Significant at *p* < .05

*p* < .01

*p* < .001.

Total *N* reduced due to the exclusion of the small number of Don’t Know responses on ethnicity and education from analysis.

Multinomial logistic regression model comparing between any use of NRT either exclusively or in combination with other aids but exclude NVP for LQA and no aid/other aids as the reference.

Multinomial logistic regression model comparing between any use of NVP either exclusively or in combination with other aids but exclude NRT for LQA and no aid/other aids as the reference.

Multinomial logistic regression model comparing between use of both NRT and NVP for LQA either exclusively or in combination with other aids and no aid/other aids as the reference.

All models adjusted for the other variable in the table, along with age, gender, income, education, ethnicity, country, knowledge of smoking harms and nicotine harm belief.

Smokers were 2.11 times as likely to use NVPs (vs. other/no aids) for quitting smoking during their LQA if they perceive that NVPs are much less harmful than cigarettes (aRRR = 2.11, 95% CI = 1.29–3.45) but were 0.53 times as likely to use NVPs (vs. other/no aids) if they did not know how harmful NVPs are relative to cigarettes (aRRR = 0.53, 95% CI = 0.29–0.96).

### Association of NRT and NVP Harm Perceptions Relative to Cigarettes with the Other Product Use (ie, Cross-product Use) as a Quit Aid during LQA

Smokers were 0.34 times as likely to use NRT for LQA (vs. other/no aids) if they perceive that NVP is much less harmful than cigarettes (aRRR = 0.34, 95% CI = 0.20–0.60) compared to those who perceive NVP is equally/more harmful than cigarettes. However, NVP use was not predicted by perceptions about NRT harmfulness.

### Country Differences in Predictive Associations

There was little statistical evidence for an interaction between country and relative harm perceptions of NRT or NVPs on use of NRT or NVPs during LQA (*p* = .24 and .21, respectively), with a similar relationship observed across all countries.

### Additional Analyses

Table 3 (see bivariate associations in Supplementary Table S3 and full details in Supplemental Table S4) shows the results of additional analyses exploring whether and how nicotine product harm perceptions might predict use of any nicotine product(s) and sole use of nonnicotine products for LQA, with no aids as the comparator. Relative to use of no aids for LQA, smokers were 2.19 times as likely to use any nicotine product(s) during LQA if they perceived that NRT is much less harmful relative to cigarettes compared to perceiving that NRT is equally/more harmful relative to cigarettes (aRRR = 2.19, 95% CI = 1.39–3.47). Use of any nicotine product(s) during LQA compared to use of no aids was not associated with NVP relative harm perceptions except for the subgroup who did not know the relative harmfulness of NVPs being 0.54 times as likely to do so (aRRR = 0.54, 95% CI = 0.34–0.86). Neither NRT nor NVP relative harm perceptions were associated with the likelihood of using only nonnicotine aids (vs. no aids) during LQA.

**Table 3.** Prospective Association of Wave 1 Nicotine Product Harm Perceptions with Wave 2 Use of Nicotine and Nonnicotine Aids for Last Quit Attempts Among Baseline Daily Smokers Smoking 10+ Cigarettes per day who had Made a Quit Attempt by Wave 2 (*N* = 1289^^^).

Wave 1 Predictors	Wave 2 any nicotine aids^#^ vs. no aids ^a^ aRRR (95% CI)	Wave 2 nonnicotine aids only vs. no aids^b^ aRRR (95% CI)
NRT Relative Harm Perception		
Much Less harmful	**2.22 (1.40, 3.52)** ^***^	0.93 (0.49, 1.73)
Somewhat Less harmful	1.47 (0.95, 2.25)	0.86 (0.48, 1.55)
Equal/More harmful	*Reference*	*Reference*
Do not know	0.97 (0.55, 1.72)	0.57 (0.25, 1.26)
NVP Relative Harm Perception		
Much Less harmful	1.12 (0.72, 1.76)	1.32 (0.69, 2.54)
Somewhat Less harmful	1.01 (0.70, 1.45)	1.14 (0.69, 1.95)
Equal/More harmful	*Reference*	*Reference*
Do not know	**0.53 (0.33, 0.84)** ^**^	0.92 (0.48, 1.74)

Note: NRT = Nicotine Replacement Therapy, NVP = Nicotine Vaping Products;

aRRR = adjusted Relative Risk Ratio, CI = Confidence Intervals;

Significant at *p* < .01

*p* < .001.

Total *N* reduced due to the exclusion of the small number of Don’t Know responses on ethnicity and education from analysis.

Include use of heated tobacco products [HTP] and smokeless tobacco (asked only in Canada and the US).

Model comparing no aid (reference) with any nicotine aids (ie, any use of NRT, NVP, HTP or smokeless tobacco, either alone or in combination with other aids for LQA).

Model comparing no aid (reference) with exclusively nonnicotine aids for LQA.

All models adjusted for the other variable in the table, along with age, gender, income, education, ethnicity, country, knowledge of smoking harms and nicotine harm belief;

## Discussion

As hypothesized, the results indicated that daily smokers’ perceptions of harm of NRT and NVPs relative to cigarettes were predictive of subsequent use of that same product. Smokers who perceived NRT as either much less or somewhat less harmful than cigarettes were more likely to use NRT for their LQA but were no more or less likely to use NVPs. Those who perceived NVPs as much less harmful were more likely to use it for their LQA. Those who did not know how harmful NVPs are, were less likely to do so. Interestingly, perceptions of NVPs being much less harmful than cigarettes also predicted a lower likelihood of NRT use at their LQA. We also found that use of both products together during their LQA was more likely if smokers perceived NRT is much less harmful than cigarettes, but they were less likely to do so if they did not know the relative harmfulness of NVPs. No country differences were observed in these relationships.

Consistent with past studies,^11,15,20^ our findings confirm that harm perceptions influence nicotine product use, with accurate perceptions associated with greater likelihood of use as a smoking cessation aid, whereas inaccurate perceptions appear to deter their use for this purpose. The finding of a weaker association between harm perception and use for NVP than for NRT is noteworthy and may suggest that harm perceptions contribute less as a determinant of product use for NVPs than for NRT. This may be due to NVP novelty and less well-established efficacy and safety as a cessation product as compared to NRT. In this regard, our finding that believing NVPs are much less harmful than cigarettes is associated with less NRT use. This suggests NVPs may be the preferred option when perceived as much less harmful than cigarettes and hence, they are considered acceptable substitutes for NRT as a smoking cessation aid.^39^

Our finding of a predictive relationship between NVP/NRT harm perceptions and their use has important implications as it suggests that use of these products for smoking cessation could be undermined by misperceptions of their harmfulness. Past research suggests that potential sources of harm misperceptions of nicotine-containing products include inaccurate beliefs about the links between nicotine and cancer,^13^ exposure to misinformation from social media, government websites and tobacco industry,^25,40^ and interpreting uncertainty about the long-term health effects of product use as indicator of significant unknown risks.^41^ Given that NRT and NVPs are the two most popular smoking cessation aids,^5^ and that there was still a substantial number of smokers who remain either misinformed or unaware of the relative harmfulness of NRT and NVPs compared to cigarettes, education and accurate messaging around the harms of these nicotine products is needed. An accurate understanding of NRT/NVP harms would ensure that smokers are able to make informed choices about whether these products are appropriate for them to use as quit aids.

The finding of cross-product influences of NVP harm perceptions on the use of NRT as an aid for smoking cessation is novel and suggests that harm perceptions of one nicotine product may influence use of another. Our study suggests that an accurate understanding of NRT harmfulness relative to cigarettes may promote not only NRT use but also other nicotine products. An accurate understanding of NVPs, on the other hand, appears to promote mainly its own use for smoking cessation. This suggestion is consistent with an accurate NRT harm perception being associated with use of any forms of nicotine aids (vs. no aids) but not that of NVP, thus providing further support for the generality of NRT harm perception effect as opposed to the more product-specific effect of NVP harm perception. Taken together, these findings suggest that the influence of NRT harm perceptions on choice of nicotine products for smoking cessation may be much less discriminating compared to that of NVPs. This could be because NVPs are perceived to possess other qualities that make them a more attractive cessation aid than NRT, such as providing better nicotine delivery and behavioral substitutability than NRT.^42^ Nevertheless, our findings require replication to confirm using other samples and across a broader range of nicotine products (eg, heated tobacco products).

The level of misperceptions of nicotine product harmfulness varied across our studied countries. We showed that smokers in England were the least, while those in the United States were the most, misinformed about the harmfulness of NRT and NVPs relative to cigarettes. This is consistent with prior research.^27^ There was little evidence of any by-country interactions for our main findings, suggesting the effect is likely related to beliefs, not to aspects of the country context although low statistical power to detect an interaction effect means this finding should be interpreted with caution. Given past efforts to educate smokers about NRT safety, the intransigence of a proportion of the smoking population to continue believing NRT is more harmful than the evidence calls for more efforts to address this issue. It is similarly important to correct any misperceptions of the harmfulness of NVPs relative to cigarettes so that smokers are not dissuaded from switching to a lower risk nicotine product, either as a short-term cessation aid^17^ or a longer-term substitute where warranted, such as for highly dependent smokers.

Past research has indicated that corrective health education can help improve smokers’ knowledge about nicotine health effects but there are challenges as well.^43^ Recent research indicates the need for absolute and comparative risk communication around nicotine and nicotine-containing products to be well-designed, evidence-based and preferably tailored to a specific target audience (eg, smokers) to avoid unintended consequences such as uptake among non-smokers.^44–46^ Pack inserts may be an efficient and effective way to educate smokers about the health harms of nicotine-containing products such as NRT.^47^ Offering a trial of NRT to all smokers including those who are reluctant to use NRT may help dispel any safety concerns they may have about NRT. This is best done by health professionals as they are in an ideal position to provide education, given that smokers are much more likely to visit a health professional than the general nonsmoking population.^48^ An advantage of providing education through pack inserts and health professionals is that these channels avoid exposure among nonsmokers, thus minimizing any unintended consequences. In many countries, NRT is the medically approved therapy for smoking cessation and is included in clinical practice guidelines.^6,7^ In countries like Australia, NVPs are cautiously recommended in clinical practice guidelines as a second line treatment for smokers to help them quit smoking.^6^ From October 1, 2021, Australian smokers can only access nicotine liquid/pods with a doctor’s prescription which can be dispensed at a local or online pharmacy.^49^ However, this option was not available at the time of the survey. While this new access arrangement could increase the opportunities for doctors and pharmacists to educate smokers about the use of NVPs for quitting smoking, including their relative harmfulness, it is currently unclear what proportion of Australian prescribers and dispensers will support the use of NVPs for smoking cessation.

### Study Strengths and Limitations

Study strengths include prospective cohort design over 2 years and cross-country comparisons. Nevertheless, several study limitations warrant some discussion. First, our survey did not assess perception of harms of NRT against NVPs. It remains unclear how any perceived differences in comparative harms of the two products might affect their use for the purpose of smoking cessation. Second, findings may be biased by the reliance on self-report data which may underestimate brief unassisted quit attempts as they are less memorable and tend to be forgotten. Third, it is unclear how participants interpreted the survey question because some may have considered addictiveness to be a harm, or they may have considered short-term side-effects of product use. Fourth, our findings predate e-cigarette or vaping use associated lung injury (EVALI), which has been shown to influence NVP harm perceptions and NVP use,^50^ and thus may not generalize beyond the study period. Relatedly, our findings may also not generalize to populations outside of the four countries studied, or to smokers who had not recently attempted to quit. Finally, although the associations were longitudinal, this of itself does not demonstrate causality given there might be other potential confounders not controlled for in our analysis, thus our causal interpretations should be treated as possibilities, not demonstrated realities.

### Conclusions

This study found that smokers’ perceptions of the harmfulness of NRT and NVPs relative to smoking predicted their respective use as an aid for smoking cessation. Accurate perceptions of NRT may further promote use of any nicotine product for smoking cessation, but accurate perceptions of NVPs may deter use of NRT. Given misperceptions of these two most popular quit aids remain unacceptably high, provision of corrective education to smokers is clearly required.

## Supplementary Material

A Contributorship Form detailing each author’s specific involvement with this content, as well as any supplementary data, are available online at https://academic.oup.com/ntr.

ntac087_suppl_Supplementary_Materials

ntac087_suppl_Supplementary_Taxonomy-form

## Data Availability

The data are jointly owned by a third party in each country that collaborates with the International Tobacco Control Policy Evaluation (ITC) Project. Data from the ITC Project are available to approved researchers 2 years after the date of issuance of cleaned data sets by the ITC Data Management Centre. Researchers interested in using ITC data are required to apply for approval by submitting an International Tobacco Control Data Repository (ITCDR) request application and subsequently to sign an ITCDR Data Usage Agreement. To avoid any real, potential, or perceived conflict of interest between researchers using ITC data and tobacco-related entities, no ITCDR data will be provided directly or indirectly to any researcher, institution, or consultant that is in current receipt of any grant monies or in-kind contribution from any tobacco manufacturer, distributor, or other tobacco-related entity. The criteria for data usage approval and the contents of the Data Usage Agreement are described online (http://www.itcproject.org). The authors of this paper obtained the data following this procedure. This is to confirm that others would be able to access these data in the same manner as the authors. The authors did not have any special access privileges that others would not have.
