# Supplementary material for: Do Smokers’ Perceptions of the Harmfulness of Nicotine Replacement Therapy and Nicotine Vaping Products as Compared to Cigarettes Influence Their Use as an Aid for Smoking Cessation? Findings from the ITC Four Country Smoking and Vaping Surveys
Source: Nicotine Tob Res. 2022 Apr 3;24(9):1413–21. doi: 10.1093/ntr/ntac087 (PMC9356684; doi:10.1093/ntr/ntac087)
Supplement: ntac087_suppl_Supplementary_Materials [file ntac087_suppl_supplementary_materials.docx]

| **4CV1 (2016) Main Sample**: n = 12,294  **Lost to follow-up at 2018 (n=2,804)**  **Excluded (n=1,756)**  No: n=1,756  **Since you completed the last survey, have you tried to stop smoking?**  Yes: n=1,315  **Study sample:** n=1,315  **Daily smokers 10+ cigarettes per day eligible for the sub-study**  (n= 5,875)  **Successfully followed up at Wave 2 (2018)**  Yes: n=3,071  **Excluded (n=6,419)**  Smoke < daily: 2,602  Smoke daily but <10 cig per day: 2,290  Quit smoking: 1,453  Non-smoker: 74  **Supplementary Figure S1.** Study Sample Flow Diagram  Supplementary Table S1. Multinomial logistic regression results showing the bivariate association of NRT harm perception, NVP harm perception and nicotine harm belief at Wave 1 with choice of nicotine product used as an aid for last quit attempts at Wave 2.   \| **Wave 1 Predictors** \| **Wave 2 NRT use**  **vs other/no aids ^a^**  **RRR (95% CI), p** \| **Wave 2 NVP use**  **vs other/no aids ^b^**  **RRR (95% CI), p** \| **Wave 2 Both NRT & NVP use**  **vs other/no aids ^c^**  **RRR (95% CI), p** \| \| --- \| --- \| --- \| --- \| \| **NRT Relative Harm Perception**  Much Less harmful  Somewhat Less harmful  Equal/More harmful  Do not know \| **2.75 (1.67, 4.54), <.001**  **1.66 (1.00, 2.75), .049**  *Reference*  0.92 (0.48, 1.77), .797 \| **2.11 (1.35, 3.32), .001**  **1.79 (1.15, 2.79), .010**  *Reference*  0.98 (0.56, 1.74), .952 \| **2.13 (1.24, 3.67), .007**  1.28 (0.74, 2.23), .378  *Reference*  **0.38 (0.16, 0.95), .037** \| \| **NVP Relative Harm Perception**  Much Less harmful  Somewhat Less harmful  Equal/More harmful  Do not know \| 0.68 (0.42, 1.12), .132  1.03 (0.71, 1.50), .864  *Reference*  0.78 (0.50, 1.20), .258 \| **2.42 (1.60, 3.66), <.001**  **1.51 (1.05, 2.19), .028**  *Reference*  **0.52 (0.32, 0.86), .010** \| **1.98 (1.19, 3.30), .009**  1.41 (0.90, 2.23), .137  Reference  **0.31 (0.15, 0.64), .002** \| \| **Nicotine Harmfulness Belief**  Not at all harmful  Slightly  Moderately  Very  Extremely  Do not know \| *Reference*  0.59 (0.23, 1.53), .277  0.55 (0.23, 1.32), .182  0.56 (0.24, 1.32), .186  0.72 (0.30, 1.71), .453  0.65 (0.20, 2.12), .475 \| *Reference*  0.62 (0.26, 1,51), .293  0.54 (0.24, 1.21), .134  0.60 (0.27, 1.34), .215  0.71 (0.31, 1.59), .401  **0.19 (0.04, 0.80), .024** \| Reference  0.98 (0.31, 3.11), .974  0.76 (0.26, 2.24), .616  0.80 (0.28, 2.35), .689  0.75 (0.25, 2.23), .599  0.33 (0.05, 1.96), .220 \|   Note: NRT = Nicotine Replacement Therapy, NVP = Nicotine Vaping Products,  RRR = Relative Risk Ratio, CI =Confidence Intervals.  **^a^** Multinomial logistic regression model comparing between any use of NRT either exclusively or in combination with other aids but exclude NVP for LQA and no aid/other aids as the reference;  **^b^** Multinomial logistic regression model comparing between any use of NVP either exclusively or in combination with other aids but exclude NRT for LQA and no aid/other aids as the reference;  ^c^ Multinomial logistic regression model comparing between use of both NRT and NVP for LQA either exclusively or in combination with other aids and no aid/other aids as the reference;  Supplementary Table S2. Multinomial logistic regression results showing the association between Wave 1 nicotine product harm perception and Wave 2 choice of nicotine product used as an aid for last quit attempts adjusting for covariates: Outcome set other/no aids as the reference for comparison.   \| **Wave 1 Predictors** \| **N (%)** \| **Wave 2 NRT use**  **vs other/no aids ^a^**  **aRRR (95% CI), p** \| **Wave 2 NVP use**  **vs other/no aids ^b^**  **aRRR (95% CI), p** \| **Both NRT & NVP use**  **vs other/no aids ^c^**  **aRRR (95% CI), p** \| \| --- \| --- \| --- \| --- \| --- \| \| **NRT Relative Harm Perception**  Much Less harmful  Somewhat Less harmful  Equal/More harmful  Do not know \| 480 (37)  502 (39)  172 (13)  135 (10) \| **3.79 (2.16, 6.66), <.001**  **1.98 (1.15, 3.42), .014**  *Reference*  0.98 (0.45, 2.11), .955 \| 1.51 (0.88, 2.61), .135  1.47 (0.89, 2.05), .132  *Reference*  1.69 (0.85, 3.36), .132 \| **1.96 (1.03, 3.73), .041**  1.16 (0.62, 2.15), .645  *Reference*  0.82 (0.29, 2.25), .695 \| \| **NVP Relative Harm Perception**  Much Less harmful  Somewhat Less harmful  Equal/More harmful  Do not know \| 264 (20)  521 (40)  297 (23)  207 (16) \| **0.34 (0.20, 0.60), <.001**  0.69 (0.46, 1.05), .087  *Reference*  0.64 (0.38, 1.07), .089 \| **2.11 (1.29, 3.45), .003**  1.34 (0.88, 2.05), .170  *Reference*  **0.53 (0.29, 0.96), .036** \| 1.29 (0.71, 2.35), .403  1.20 (0.72, 2.01), .477  Reference  **0.32 (0.14, 0.75), .009** \| \| **Nicotine Harmfulness Belief**  Not at all harmful  Slightly  Moderately  Very  Extremely  Do not know \| 40 (3)  129 (10)  334 (26)  436 (34)  322 (25)  28 (2) \| *Reference*  0.63 (0.23, 1.75), .379  0.52 (0.20, 1.36), .184  0.47 (0.18, 1.22), .119  0.64 (0.24, 1.70), .373  0.97 (0.27, 3.57), .969 \| *Reference*  0.74 (0.29, 1,92), .535  0.80 (0.33, 1.93), .617  0.78 (0.32, 1.88), .578  0.83 (0.34, 2.06), .689  0.36 (0.08, 1.72), .203 \| Reference  1.13 (0.34, 3.78), .841  0.92 (0.29, 2.90), .885  0.86 (0.27, 2.70), .791  0.80 (0.25, 2.61), .714  0.77 (0.12, 5.12), .789 \| \| **Country**  Canada  United States  England  Australia \| 448 (35)  219 (17)  342 (27)  280 (22) \| *Reference*  **0.51 (0.32, 0.82), .006**  **0.61 (0.39, 0.96), .033**  0.77 (0.52, 1.15), .204 \| *Reference*  0.81 (0.52, 1.26), .349  **2.19 (1.50, 3.20), <.001**  **0.54 (0.35, 0.85), .007** \| Reference  **0.47 (0.27, 0.82), .008**  0.74 (0.46, 1.20), .224  **0.39 (0.23, 0.67), .001** \| \| **Age Group**  18-24  25-39  40-54  55+ \| 82 (6)  236 (18)  428 (33)  543 (42) \| *Reference*  0.90 (0.37, 2.19), .824  1.80 (0.79, 4.09), .163  1.72 (0.52, 1.15), .189 \| *Reference*  0.84 (0.45, 1.57), .595  0.91 (0.50, 1.64), .748  **0.46 (0.25, 0.83), .010** \| Reference  1.14 (0.50, 2.60), .749  1.28 (0.58, 2.83), .540  0.85 (0.38, 1.86), .677 \| \| **Gender**  Male  Female \| 606 (47)  683 (53) \| *Reference*  1.20 (0.87, 1.65), .256 \| *Reference*  1.22 (0.91, 1.66), .187 \| Reference  **1.59 (1.09, 2.32), .017** \| \| **Education**  Low  Moderate  High \| 473 (37)  537 (42)  279 (22) \| *Reference*  0.94 (0.66, 1.34), .737  1.04 (0.67, 1.59), .871 \| *Reference*  1.36 (0.97, 1.90), .074  0.90 (0.59, 1.38), .639 \| Reference  1.06 (0.69, 1.61), .803  1.14 (0.69, 1.90), .605 \| \| **Income**  Low  Moderate  High  No information \| 420 (33)  421 (33)  377 (29)  71 (6) \| *Reference*  0.97 (0.65, 1.45), .885  1.02 (0.67, 1.54), .932  1.20 (0.61, 2.33), .601 \| *Reference*   - 1. (0.70, 1.46), .961   0.96 (0.64, 1.43), .831  0.60 (0.29, 1.25), .175 \| Reference  0.96 (0.61, 1.52), .861  0.91 (0.55, 1.48), .698  0.54 (0.21, 1.42), .211 \| \| **Ethnicity**  Ethnic majority  Ethnic minority \| 1186 (92)  103 (8) \| *Reference*  1.02 (0.55, 1.89), .958 \| *Reference*  1.07 (0.62, 1.85), .795 \| Reference  1.01 (0.51, 2.01), .982 \| \| **Knowledge of Smoking Harms** \| 1289 (100) \| 1.14 (0.99, 1.32), .077 \| **1.19 (1.02, 1.37), .018** \| 1.10 (0.92, 1.31), .299 \|   Note: NRT = Nicotine Replacement Therapy, NVP = Nicotine Vaping Products, aRRR = adjusted Relative Risk Ratio, CI = Confidence Intervals.  **^a^** Multinomial logistic regression model comparing between any use of NRT either exclusively or in combination with other aids but exclude NVP for LQA and no aid/other aids as the reference;  **^b^** Multinomial logistic regression model comparing between any use of NVP either exclusively or in combination with other aids but exclude NRT for LQA and no aid/other aids as the reference;  ^c^ Multinomial logistic regression model comparing between use of both NRT and NVP for LQA either exclusively or in combination with other aids and no aid/other aids as the reference;  All models adjusted for the other variable in the table, along with age, gender, income, education, ethnicity, country, knowledge of smoking harms and nicotine harm belief; |
| --- | --- | --- | --- | --- | --- | --- | --- | --- | --- | --- | --- | --- | --- | --- | --- | --- | --- | --- | --- | --- | --- | --- | --- | --- | --- | --- | --- | --- | --- | --- | --- | --- | --- | --- | --- | --- | --- | --- | --- | --- | --- | --- | --- | --- | --- | --- | --- | --- | --- | --- | --- | --- | --- | --- | --- | --- | --- | --- | --- | --- | --- | --- | --- | --- | --- | --- | --- | --- | --- | --- | --- |

Supplementary Table S3. Multinomial logistic regression results showing the bivariate association of NRT harm perception, NVP harm perception and nicotine harm belief at Wave 1 with choice of nicotine and non-nicotine aids for last quit attempts at Wave 2 (N=1315).

| **Wave 1 Predictors** | **Any nicotine aids at Wave 2**  **vs no aids ^a,^ ^**  **RRR (95% CI), p** | **Non-nicotine aids only at Wave 2 vs no aids ^b^**  **RRR (95% CI), p** |
| --- | --- | --- |
| **NRT Relative Harm Perception**  Much Less harmful  Somewhat Less harmful  Equal/More harmful  Do not know | **2.29 (1.54, 3.40), <.001**  **1.55 (1.05, 2.27), .026**  *Reference*  0.67 (0.41, 1.08), .100 | 1.08 (0.63, 1.86), .777  0.98 (0.58, 1.65), .927  *Reference*  0.58 (0.29, 1.15), .118 |
| **NVP Relative Harm Perception**  Much Less harmful  Somewhat Less harmful  Equal/More harmful  Do not know | **1.72 (1.16, 2.54), .007**  1.26 (0.91, 1.74), .162  *Reference*  **0.54 (0.37, 0.79), .002** | 1.32 (0.75, 2.32), .341  1.05 (0.65, 1.69), .848  *Reference*  0.89 (0.52, 1.52), .656 |
| **Nicotine Harmfulness Belief**  Not at all harmful  Slightly  Moderately  Very  Extremely  Do not know | *Reference*  0.54 (0.23, 1.30), .171  0.50 (0.22, 1.14), .098  0.57 (0.25, 1.28), .175  0.72 (0.32, 1.65), .436  0.34 (0.11, 1.04), .058 | *Reference*  0.43 (0.12, 1.58), .202  0.59 (0.18, 1.90), .374  0.76 (0.24, 2.41), .637  1.01 (0.31, 3.27), .983  0.73 (0.16, 3.39), .685 |

Note: NRT = Nicotine Replacement Therapy, NVP = Nicotine Vaping Products,

RRR = Relative Risk Ratio, CI = Confidence Intervals.

^ include use of heated tobacco products and smokeless tobacco (asked only in Canada and the US);

^a^ Model comparing no aid (reference) with any nicotine aids (i.e., any use of NRT, NVP, HTP or smokeless tobacco, either alone or in combination with other aids for LQA;

^b^ Model comparing no aid (reference) with exclusively non-nicotine aids for LQA;

Supplementary Table S4. Multinomial logistic regression results showing the association between Wave 1 nicotine product harm perception and Wave 2 choice of nicotine and non-nicotine aids for last quit attempts: Outcome set no aids as the reference for comparison (N=1289^#^).

| **Wave 1 Predictors** | **N (%)** | **Any nicotine aids at Wave 2**  **vs no aids ^a,^ ^**  **aRRR (95% CI), p** | **Non-nicotine aids only at Wave 2**  **vs no aids ^b^**  **aRRR (95% CI), p** |
| --- | --- | --- | --- |
| **NRT Relative Harm Perception**  Much Less harmful  Somewhat Less harmful  Equal/More harmful  Do not know | 480 (37)  502 (39)  172 (13)  135 (10) | **2.22 (1.40, 3.52), .001**  1.47 (0.95, 2.25), .081  *Reference*  0.97 (0.55, 1.72), .921 | 0.93 (0.49, 1.73), .807  0.86 (0.48, 1.55), .623  *Reference*  0.57 (0.25, 1.26), .165 |
| **NVP Relative Harm Perception**  Much Less harmful  Somewhat Less harmful  Equal/More harmful  Do not know | 264 (20)  521 (40)  297 (23)  207 (16) | 1.12 (0.72, 1.76), .610  1.01 (0.70, 1.45), .967  *Reference*  **0.53 (0.33, 0.84), .007** | 1.32 (0.69, 2.54), .398  1.14 (0.69, 1.95), .624  *Reference*  0.92 (0.48, 1.74), .786 |
| **Nicotine Harmfulness Belief**  Not at all harmful  Slightly  Moderately  Very  Extremely  Do not know | 40 (3)  129 (10)  334 (26)  436 (34)  322 (25)  28 (2) | *Reference*  0.64 (0.25, 1.59), .333  0.63 (0.26, 1.51), .297  0.64 (0.26, 1.53), .312  0.84 (0.34, 2.06), .698  0.73 (0.22, 2.46), .609 | *Reference*  0.42 (0.11, 1.59), .200  0.52 (0.15, 1.78), .299  0.68 (0.19, 2.30), .529  1.02 (0.29, 3.55), .977  0.95 (0.19, 4.78), .946 |
| **Country**  Canada  United States  England  Australia | 448 (35)  219 (17)  342 (27)  280 (22) | *Reference*  **0.55 (0.37, 0.81), .002**  0.97 (0.68, 1.38), .866  **0.64 (0.45, 0.93), .018** | *Reference*  0.76 (0.44, 1.31), .324  0.56 (0.32, 0.98), .043  1.21 (0.74, 1.97), .741 |
| **Age Group**  18-24  25-39  40-54  55+ | 82 (6)  236 (18)  428 (33)  543 (42) | *Reference*  0.83 (0.46, 1.52), .551  1.29 (0.73, 2.29), .384  0.83 (0.48, 1.46), .522 | *Reference*  1.11 (0.39, 3.13), .845  2.04 (0.76, 5.46), .155  1.67 (0.63, 4.41), .302 |
| **Gender**  Male  Female | 606 (47)  683 (53) | *Reference*  **1.49 (1.14, 1.95), .003** | *Reference*  **1.78 (1.21, 2.63), .003** |
| **Education**  Low  Moderate  High | 473 (37)  537 (42)  279 (22) | *Reference*  1.07 (0.79, 1.44), .681  0.95 (0.66, 1.37), .779 | *Reference*  0.90 (0.58, 1.38), .618  0.94 (0.56, 1.57), .801 |
| **Income**  Low  Moderate  High  No information | 420 (33)  421 (33)  377 (29)  71 (6) | *Reference*  1.26 (0.90, 1.77), .171  1.09 (0.77, 1.54), .640  0.80 (0.44, 1.44), .458 | *Reference*  **1.91 (1.18, 3.08), .008**  1.36 (0.82, 2.27), .235  1.03 (0.43, 2.45), .945 |
| **Ethnicity**  Ethnic majority  Ethnic minority | 1186 (92)  103 (8) | *Reference*  0.99 (0.61, 1.61), .971 | *Reference*  0.98 (0.49, 1.99), .964 |
| **Knowledge of Smoking Harms** | 1289 (100) | 1.11 (0.98, 1.25), .107 | 0.92 (0.76, 1.09), .340 |

Note: NRT = Nicotine Replacement Therapy, NVP = Nicotine Vaping Products,

aRRR = adjusted Relative Risk Ratio, CI = Confidence Intervals.

^ include use of heated tobacco products and smokeless tobacco (asked only in Canada and the US);

^#^ Total N reduced due to the exclusion from analysis of the small number of Don’t Know responses on ethnicity and education.

^a^ Model comparing no aid (reference) with any nicotine aids (i.e., any use of NRT, NVP, HTP or smokeless tobacco, either alone or in combination with other aids for LQA;

^b^ Model comparing no aid (reference) with exclusively non-nicotine aids for LQA;

All models adjusted for the other variable in the table, along with age, gender, income, education, ethnicity, country, knowledge of smoking harms and nicotine harm belief;
