# Supplementary material for: Do Smokers’ Perceptions of the Harmfulness of Nicotine Replacement Therapy and Nicotine Vaping Products as Compared to Cigarettes Influence Their Use as an Aid for Smoking Cessation? Findings from the ITC Four Country Smoking and Vaping Surveys
Source: Nicotine Tob Res. 2022 Apr 3;24(9):1413–21. doi: 10.1093/ntr/ntac087 (PMC9356684; doi:10.1093/ntr/ntac087)
Supplement: ntac087_suppl_Supplementary_Taxonomy-form [file ntac087_suppl_supplementary_taxonomy-form.pdf]

Contributorship form for publication in *Nicotine & Tobacco Research*

Authorship Contribution

In submitting this manuscript to *Nicotine & Tobacco Research*, the corresponding author and any co-authors are confirming that each and every author has, in accordance with the ICMJE guidelines, (a) made a substantial contribution to the conception and design, acquisition of data, and/or analysis and interpretation of data, (b) participated in the drafting of the article or revising it critically for important intellectual content, (c) have read and approved the final manuscript, AND (d) that the manuscript is not, either in whole or in part, currently under consideration by any other scientific journal and has not been previously published in either hard copy or electronic format. Except for those submitting the manuscript, members of Study Groups should not be individually cited as authors; instead the Study Group should be named as an author, and individuals within the group listed in full in an acknowledgement section of the manuscript.

This journal also requires that each and every author's specific contributions to the manuscript be described using the Contributor Roles Taxonomy (CRediT). This taxonomy includes 14 descriptors representing the roles typically played by contributors to scientific scholarly outputs, and the roles describe each contributor's specific contributions to the manuscript. **For accepted manuscripts, this completed form will publish online as supplementary material.**

Required Information

Manuscript Reference (e.g. NTR-2019-0378.R2): NTR-2021-864

Manuscript Title: Do smokers' perceptions of the harmfulness of nicotine replacement therapy and nicotine vaping products as compared to cigarettes influence their use as an aid for smoking cessation? Findings from the ITC Four Country Smoking and Vaping Surveys

Corresponding Author: Hua Yong

Number of Authors: 11

A detailed explanation defining the contribution of each author to the manuscript must be made using the table below.

Corresponding Author

As the corresponding author by signing this authorship form you confirm on behalf of yourself and all authors on the paper that this manuscript complies with the authorship policies of *Nicotine & Tobacco Research*.

Full First and Surname: Hua Yong

Signature: **Date: 17/02/2022**

Declaration

The corresponding author must state the contribution of *all authors* to the manuscript. If there are more than 20 co-authors, please add additional rows to the table below.

	Author Name	Conceptualization	Data Curation	Formal Analysis	Funding Acquisition	Investigation	Methodology	Project Administration	Resources	Software	Supervision	Validation	Visualization	Writing – Original Draft	Writing – Review & Editing
1	Hua-Hie Yong	☒	☒	☒	☐	☒	☒	☒	☒	☐	☒	☒	☒	☒	☒
2	Shannon Gravely	☒	☐	☐	☐	☒	☒	☐	☐	☐	☐	☐	☐	☐	☒
3	Ron Borland	☒	☐	☐	☒	☒	☒	☒	☒	☐	☐	☐	☐	☐	☒
4	Coral Gartner	☐	☐	☐	☐	☒	☒	☒	☐	☐	☐	☐	☐	☐	☒
5	K. Michael Cummings	☒	☐	☐	☒	☒	☒	☒	☒	☐	☐	☐	☐	☐	☒
6	Katherine East	☐	☐	☐	☐	☒	☒	☐	☐	☐	☐	☐	☐	☐	☒
7	Scott Tagliaferri	☐	☒	☒	☐	☐	☐	☐	☐	☐	☐	☒	☒	☒	☒
8	Tara Elton-Marshall	☐	☐	☐	☐	☒	☒	☐	☐	☐	☐	☐	☐	☐	☒
9	Andrew Hyland	☒	☐	☐	☐	☒	☒	☐	☐	☐	☐	☐	☐	☐	☒
10	Maansi Bansal-Travers	☐	☐	☐	☐	☒	☒	☐	☐	☐	☐	☐	☐	☐	☒
11	Geoffrey T. Fong	☒	☐	☐	☒	☒	☒	☒	☒	☐	☐	☐	☐	☐	☒
12	Click here to enter text.	☐	☐	☐	☐	☐	☐	☐	☐	☐	☐	☐	☐	☐	☐
13	Click here to enter text.	☐	☐	☐	☐	☐	☐	☐	☐	☐	☐	☐	☐	☐	☐
14	Click here to enter text.	☐	☐	☐	☐	☐	☐	☐	☐	☐	☐	☐	☐	☐	☐
15	Click here to enter text.	☐	☐	☐	☐	☐	☐	☐	☐	☐	☐	☐	☐	☐	☐
16	Click here to enter text.	☐	☐	☐	☐	☐	☐	☐	☐	☐	☐	☐	☐	☐	☐
17	Click here to enter text.	☐	☐	☐	☐	☐	☐	☐	☐	☐	☐	☐	☐	☐	☐
18	Click here to enter text.	☐	☐	☐	☐	☐	☐	☐	☐	☐	☐	☐	☐	☐	☐
19	Click here to enter text.	☐	☐	☐	☐	☐	☐	☐	☐	☐	☐	☐	☐	☐	☐
20	Click here to enter text.	☐	☐	☐	☐	☐	☐	☐	☐	☐	☐	☐	☐	☐	☐
